# Sulf1 and Sulf2 Differentially Modulate Heparan Sulfate Proteoglycan Sulfation during Postnatal Cerebellum Development: Evidence for Neuroprotective and Neurite Outgrowth Promoting Functions

**DOI:** 10.1371/journal.pone.0139853

**Published:** 2015-10-08

**Authors:** Ina Kalus, Susanne Rohn, Tania M. Puvirajesinghe, Scott E. Guimond, Pieter J. Eyckerman-Kölln, Gerdy ten Dam, Toin H. van Kuppevelt, Jeremy E. Turnbull, Thomas Dierks

**Affiliations:** 1 Fakultät für Chemie, Biochemie I, Universität Bielefeld, Bielefeld, Germany; 2 Centre for Glycobiology, School of Biological Sciences, University of Liverpool, Liverpool, United Kingdom; 3 Department of Biochemistry, University of Nijmegen, Nijmegen, The Netherlands, Center for Molecular Life Sciences, Radboud University Nijmegen Medical Center, Nijmegan, The Netherlands; Columbia University, UNITED STATES

## Abstract

**Introduction:**

Sulf1 and Sulf2 are cell surface sulfatases, which remove specific 6-O-sulfate groups from heparan sulfate (HS) proteoglycans, resulting in modulation of various HS-dependent signaling pathways. Both Sulf1 and Sulf2 knockout mice show impairments in brain development and neurite outgrowth deficits in neurons.

**Methodology and Main Findings:**

To analyze the molecular mechanisms behind these impairments we focused on the postnatal cerebellum, whose development is mainly characterized by proliferation, migration, and neurite outgrowth processes of precursor neurons. Primary cerebellar granule cells isolated from Sulf1 or Sulf2 deficient newborns are characterized by a reduction in neurite length and cell survival. Furthermore, Sulf1 deficiency leads to a reduced migration capacity. The observed impairments in cell survival and neurite outgrowth could be correlated to Sulf-specific interference with signaling pathways, as shown for FGF2, GDNF and NGF. In contrast, signaling of Shh, which determines the laminar organization of the cerebellar cortex, was not influenced in either Sulf1 or Sulf2 knockouts. Biochemical analysis of cerebellar HS demonstrated, for the first time in vivo, Sulf-specific changes of 6-O-, 2-O- and N-sulfation in the knockouts. Changes of a particular HS epitope were found on the surface of Sulf2-deficient cerebellar neurons. This epitope showed a restricted localization to the inner half of the external granular layer of the postnatal cerebellum, where precursor cells undergo final maturation to form synaptic contacts.

**Conclusion:**

Sulfs introduce dynamic changes in HS proteoglycan sulfation patterns of the postnatal cerebellum, thereby orchestrating fundamental mechanisms underlying brain development.

## Introduction

**Fig 1:**
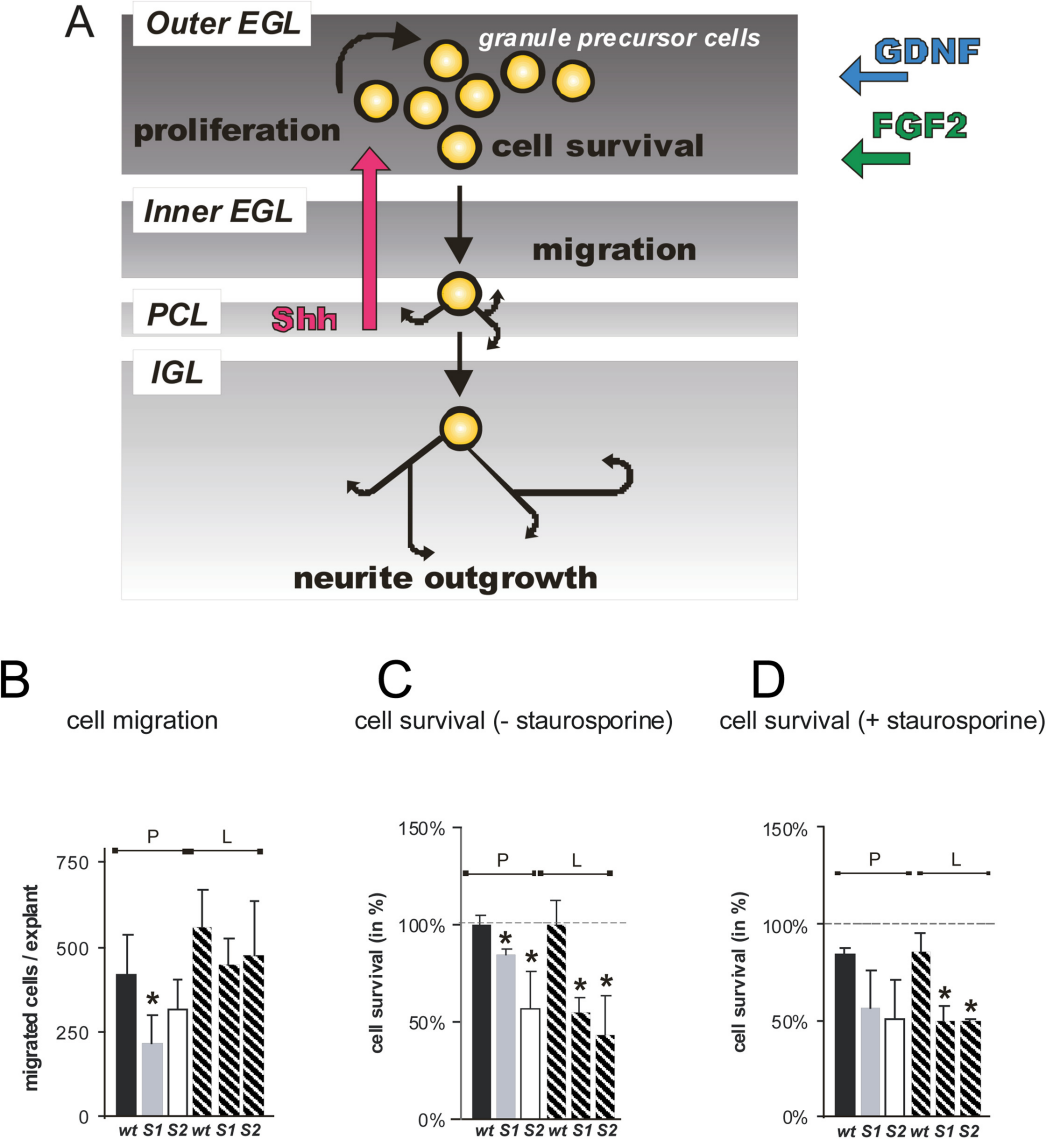
Sulf deficiency impairs the postnatal development of the cerebellum. | (**A**) The postnatal cerebellar development is mainly characterized by proliferation, migration and neurite outgrowth of granule precursor cells[1,2]. The coordination and regulation of these processes involve growth factors such as Shh, FGF2, NGF and GDNF in the local environment of these precursor neurons (for details see Introduction). (**B**) Poly-L-lysine dependent migration of Sulf1 deficient cerebellar granule cells is reduced. Cerebellar microexplant cultures from wildtype (wt), Sulf1 (S1) or Sulf2 (S2) deficient mice were plated onto glass cover slips coated with PLL (P, filled bars) or a combination of PLL and laminin (L, hatched bars). Explants were fixed, stained with DAPI and migration of cerebellar granule cells quantitated as described in Experimental Procedures. The asterisk indicates a statistically significant difference of Sulf deficient neurons as compared to wildtype cells (* p < 0.05). (**C, D**) Sulf1 and Sulf2 deficient cerebellar neurons show significant reduction of cell survival. Cerebellar neurons from wildtype (wt), Sulf1 (S1) and Sulf2 (S2) deficient mice were plated as single cell suspensions onto PLL coated cover slips (P, filled bars) or cover slips coated with a combination of PLL and laminin (L, hatched bars). For cells shown in D, 44 hours after plating 500 nM staurosporine was added to the medium to induce cell death. The cells were cultured for additional 4 h with (D) or without (C) staurosporine before cell death was determined by counting calcein versus propidium iodide positive cells of five independent experiments. Cell survival of wildtype cells cultured without staurosporine was set to 100%. Asterisks indicate a statistically significant difference of Sulf deficient neurons as compared to wildtype cells (* p < 0.05).

The development of the postnatal cerebellar cortex is mainly characterized by proliferation, migration and neurite outgrowth of granule precursor cells [1,2]. The coordination and regulation of these processes involves a complex pattern of guidance cues in the local environment of these precursor neurons. Among these cues are chemoattractants and growth promoting molecules, such as growth factors of the Shh, FGF and GDNF families which are involved in the establishment of attractive or repellent chemokine gradients and bind to cell surface receptors to initiate growth modulating signal transduction processes [3,4,5,6,7,8]. The postnatal cerebellar cortex is organized from outside to the center by the so-called i) external granular layer (EGL), further divided in an outer (oEGL) and inner half (iEGL), ii) the Purkinje cell layer (PCL) and iii) the internal granular layer (IGL) (Fig 1A). The outer half of the external granular layer (EGL) is the zone where precursor cells actively proliferate to generate a pool of later granule cells. The subjacent Purkinje cells induce the proliferation of granule cells by secreting the growth factor Shh [3,4,5], a process which has further been shown to be modulated by FGF2 as well as GDNF [7,8]. As granule precursor cells enter the inner half of the EGL, they stop to divide and start their final steps of maturation; they undergo neurite extension and tangential migration from the EGL through the Purkinje cell layer (PCL) to reach their final destination in the internal granular layer (IGL), the later granular layer [1,2].

The activities of the growth factors Shh, FGF2 and GDNF are influenced by heparan sulfate proteoglycans (HSPGs), which occur as extracellular matrix components and as membrane-anchored cell surface receptors [9,10,11]. HSPGs interact with growth factors via their HS side chains, thereby either capturing these factors or presenting them to their cognate receptors. The molecular encounter of growth factors and HS is not a static but rather a highly dynamic process, based on controlled biosynthesis of variant HS structures with differing functional activities, as demonstrated in the developing mouse neuroepithelium [12,13]. Recently, additional major regulators of this process have been discovered, namely the extracellular 6-O-endosulfatases Sulf1 and Sulf2 [14,15,16,17,18,19]. These enzymes are able to remove specific 6-O-sulfate groups from HS chains, thereby directly inhibiting or promoting different growth factor signaling pathways such as Shh, FGF-2 and GDNF [14,19,20,21,22,23,24,25], which are involved in postnatal cerebellar development.

As shown previously, the knockout of Sulf1 or Sulf2 in mice results in an impairment of behavioral and synaptic plasticity in adult animals [26]. The importance of HSPG modulation by Sulf1 and Sulf2 was further reflected by a reduced synapse density in the hippocampus and by neurite outgrowth deficits of primary cerebellar granule cells and hippocampal neurons isolated from the knockout mice [26]. To approach the molecular mechanisms behind these impairments we focused our studies on the cerebellum and addressed the following questions. i) Are these enzymes involved in basic processes underlying postnatal development like precursor cell migration or apoptosis? ii) Does impaired signal transduction contribute to the observed reduction in neurite length and other cellular deficits? iii) Do both enzymes fulfill redundant functions or contribute differentially to the observed phenotype, in terms of both functional effects and HS molecular structure? To answer these questions cell survival, migration and neurite outgrowth capacity of cerebellar granule neurons isolated from Sulf1 or Sulf2 single knockout mice under the influence of Shh, FGF2, GDNF and NGF were analyzed. In this study we focused on primary cell cultures isolated from postnatal day 6, as it was shown that the sulfation of newly synthesized HS in the cerebellum was highest at this point of development [27]. To further correlate the observed phenotypical deficits with differences in HS molecular structure, Sulf-specific changes in HS sulfation patterning were determined *in vivo* (for the postnatal cerebellum) and *in vitro* (for cerebellar neurons).

We show here that cerebellar granule cells isolated from Sulf1 or Sulf2 deficient newborns are characterized by neurite outgrowth, cell survival and migration deficits. These cellular deficits could be correlated with HSPG modulated signaling pathways. Furthermore, we demonstrate, for the first time *in vivo*, Sulf-specific changes in HS sulfation patterns in the cerebellum. Together these data strongly suggest that the observed sulfatase-specific *in vivo* phenotypes in Sulf knockout mice result from a complex mixture of molecular mechanisms, which are operative in different cell types, involve various growth promoting factors, and are orchestrated by dynamically regulated sulfation patterns of extracellular HSPGs.

## Results

### Sulf1 and Sulf2 deficient cerebellar neurons are characterized by impaired neurite outgrowth, enhanced cell death and migration deficits

The postnatal development of the cerebellar cortex is mainly characterized by proliferation, neurite outgrowth and migration of cerebellar granule cells. When isolated from Sulf1 or Sulf2 deficient mice, these primary cells exhibit a significant reduction in neurite length of about 20–30% with Sulf2, but not Sulf1 deficient cells showing this reduction specifically for laminin dependent neurite outgrowth [26]. Here we further analyzed the migration of cerebellar microexplants and the cell survival capacity of cerebellar granule cells isolated from Sulf1^-/-^, Sulf2^-/-^ and wildtype newborns. Cells were grown on the same two substrates as before [26], namely poly-L-lysine (PLL) or a combination of PLL plus the HS-dependent extracellular matrix molecule laminin (Fig 1B–1D) [28]. The total number of migrated cerebellar granule cells from microexplants of Sulf1 deficient newborns was significantly reduced for explants grown on PLL. In comparison, laminin dependent migration was not affected in case of either Sulf1 or Sulf2 deficient cerebellar granule cells (Fig 1B).

When determining the cell survival of dissociated cerebellar granule cells, a significant reduction was detectable for both Sulf1 and Sulf2 deficient neurons (Fig 1C). Moreover, this reduced cell survival was observed on either substrate, PLL with and without laminin. It had been shown earlier that hepatocellular cancer cells, expressing only low levels of Sulf1, are characterized by an insensitivity against staurosporine-induced apoptosis [29,30]. Thus, we investigated the survival of cerebellar granule cells after the induction of cell death by addition of staurosporine. Staurosporine is a broad-spectrum kinase inhibitor that is widely used as a proapoptotic stimulus. Surprisingly, not only Sulf1 but also Sulf2 deficient cerebellar neurons were both insensitive to staurosporine-induced apoptosis, whereas staurosporine enhanced cell death in wildtype neurons by about 20%, which agrees with earlier studies [31] (Fig 1, compare D and C). Clearly, despite the lack of the protective Sulfs (Fig 1C), staurosporine did not potentiate cell death in knockout cells (Fig 1D). In summary, Sulf1 and Sulf2 deficiency results in impaired neurite outgrowth and enhanced cell death of primary cerebellar neurons. In addition, lack of Sulf1 enzyme function leads to a migration deficit of cerebellar granule cells.

### Neurite outgrowth deficits and enhanced cell death of Sulf deficient cerebellar granule cells involve growth factor dependent signal transduction processes

**Fig 2:**
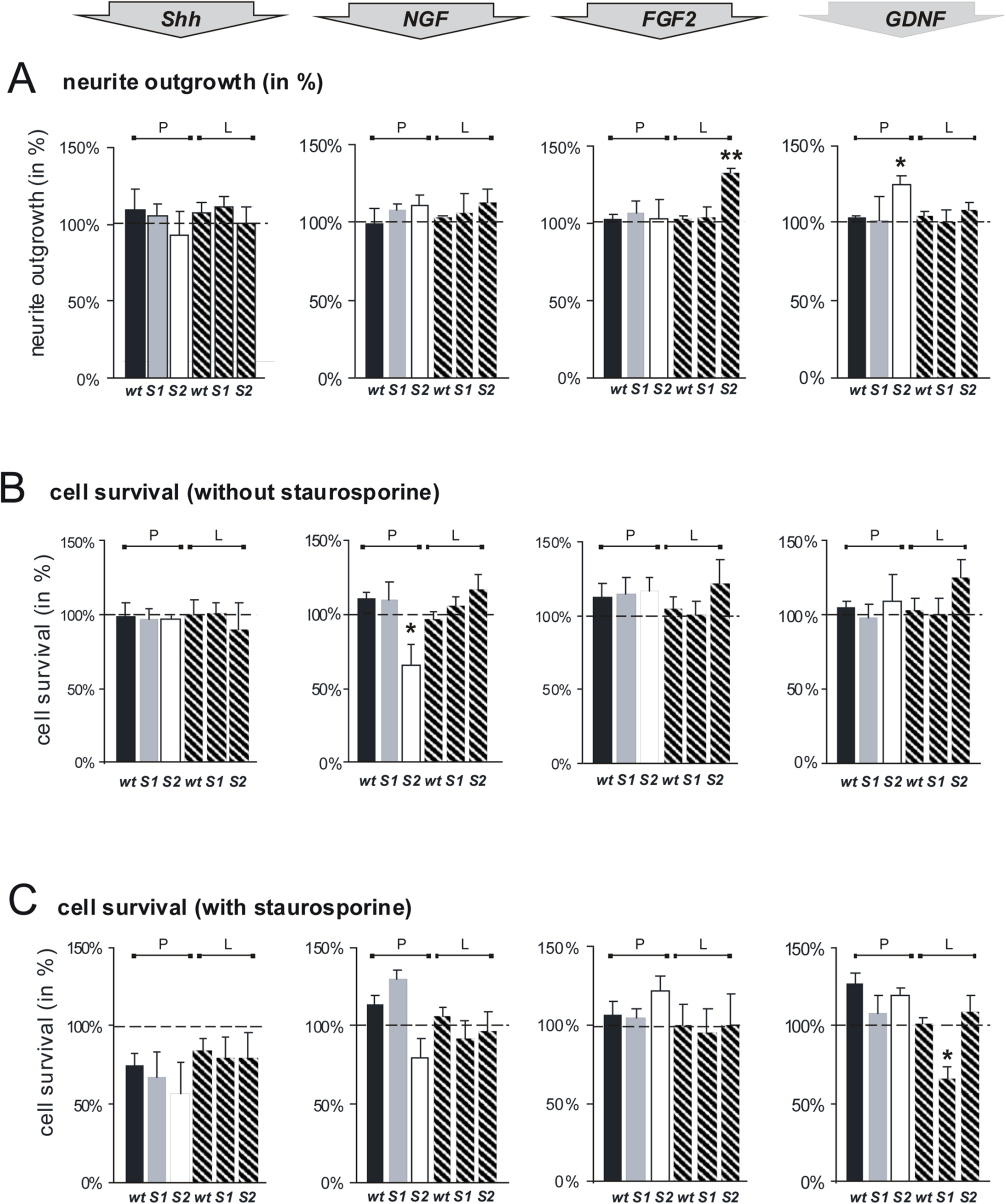
Sulf1 and Sulf2 differentially modulate NGF, FGF2 and GDNF signaling pathways and thereby influence neurite outgrowth and cell survival of cerebellar granule cells. (**A**) Cerebellar microexplant cultures from wildtype (wt), Sulf1 (S1) and Sulf2 (S2) deficient mice were plated onto glass cover slips coated with PLL (P, filled bars) or a combination of PLL and laminin (L, hatched bars). Following incubation for 24 h at 37°C with 10 ng/ml Shh, 50 ng/ml FGF 2, 10 ng/ml GDNF or 50 ng/ml NGF, explants were fixed and stained. Neurite outgrowth was quantitated by measuring the ten longest neurites of ten aggregates in three independent experiments. Neurite length of explants of each genotype cultured without any additives was set to 100%. Asterisks indicate a statistically significant difference (** p< 0.01, * p< 0.05). (**B, C**) Cerebellar neurons from wildtype (wt), Sulf1 (S1) and Sulf2 (S2) deficient mice were plated as single cell suspensions onto PLL coated cover slips (P, filled bars) or cover slips coated with a combination of PLL and laminin (L, hatched bars). Following incubation of growth factors (see A), cell death was determined by counting calcein versus propidium iodide positive cells (**B**). (**C**) 20 hours after growth factor treatment 500 nM staurosporine was added to the medium and the cells were cultured for additional 4 h. Then cell death was determined (five independent experiments). Cell survival of each genotype without any additives, see data shown in Fig 1C and 1D, was set to 100% (indicated by dashed lines). Thus, the data show the specific effects of the tested growth factors on cell survival separately for each genotype (and not in relation to wildytpe). Asterisks indicate a statistically significant difference (* p< 0.05).

Growth factors like NGF, FGF2 or GDNF promote the outgrowth and navigation of neurons [32,33]. By removing 6-O-sulfate groups from HS side chains of HSPGs, the endosulfatases Sulf1 and Sulf2 are able to modulate various signal transduction processes, amongst others those of FGF2, GDNF and Shh [14,19,21,29]. Therefore, the question arose whether the observed deficits in neurite outgrowth and cell survival are based on interference with these signal transduction pathways. We thus analyzed the effect of the growth factors Shh, FGF2, GDNF and, in addition, of the axon outgrowth stimulating factor NGF, on neurite outgrowth, cell survival and staurosporine-induced cell death of cerebellar granule cells. Fig 2 summarizes the results obtained showing the response of the cells to these growth factors relative to untreated control cells, which for each genotype were analyzed in parallel.

All tested growth factors showed only minor effects on neurite outgrowth of wildtype and Sulf1 knockout granule cells (Fig 2A). In contrast, Sulf2 deficient cerebellar explants grown on the substrate combination PLL plus laminin under the influence of FGF2 showed an enhancement of neurite length of approximately 30% (Fig 2A). In addition, neurite outgrowth from Sulf2 deficient explants was significantly enhanced under the influence of GDNF when maintained on PLL (Fig 2A).

Shh and FGF2 showed no genotype-specific differences in their effect on cell survival and staurosporine induced cell-death of dissociated cerebellar cells isolated from wildtype, Sulf1 or Sulf2 deficient animals (Fig 2B and 2C). Interestingly, the observed reduction in cell survival of granule cells maintained on the substrate PLL (Fig 1C) was significantly reduced further for Sulf2 deficient cells in the presence of NGF (Fig 2B). In addition, a similar effect was observed for Sulf1 deficient cerebellar granule cells when grown on PLL plus laminin under the influence of GDNF, which resulted in a significant increase of staurosporine-induced cell death (Fig 2C).

In summary, neurite outgrowth and cell survival deficits due to Sulf1 or Sulf2 deficiency very likely involve altered signal transduction, as shown here for the heparan sulfate dependent growth factors FGF2 and GDNF with specific effects resulting from deficiency of each of the two sulfatases. Interestingly, the influence of NGF on cell survival, known for a long time as neuroprotective growth factor (for review see [34]), is severely counteracted by loss of Sulf2, but not Sulf 1, enzyme function (Fig 2C).

### Sulf1 and Sulf2 deficiency results in sulfatase-specific differences in HS sulfation patterns *in vivo*

**Fig 3:**
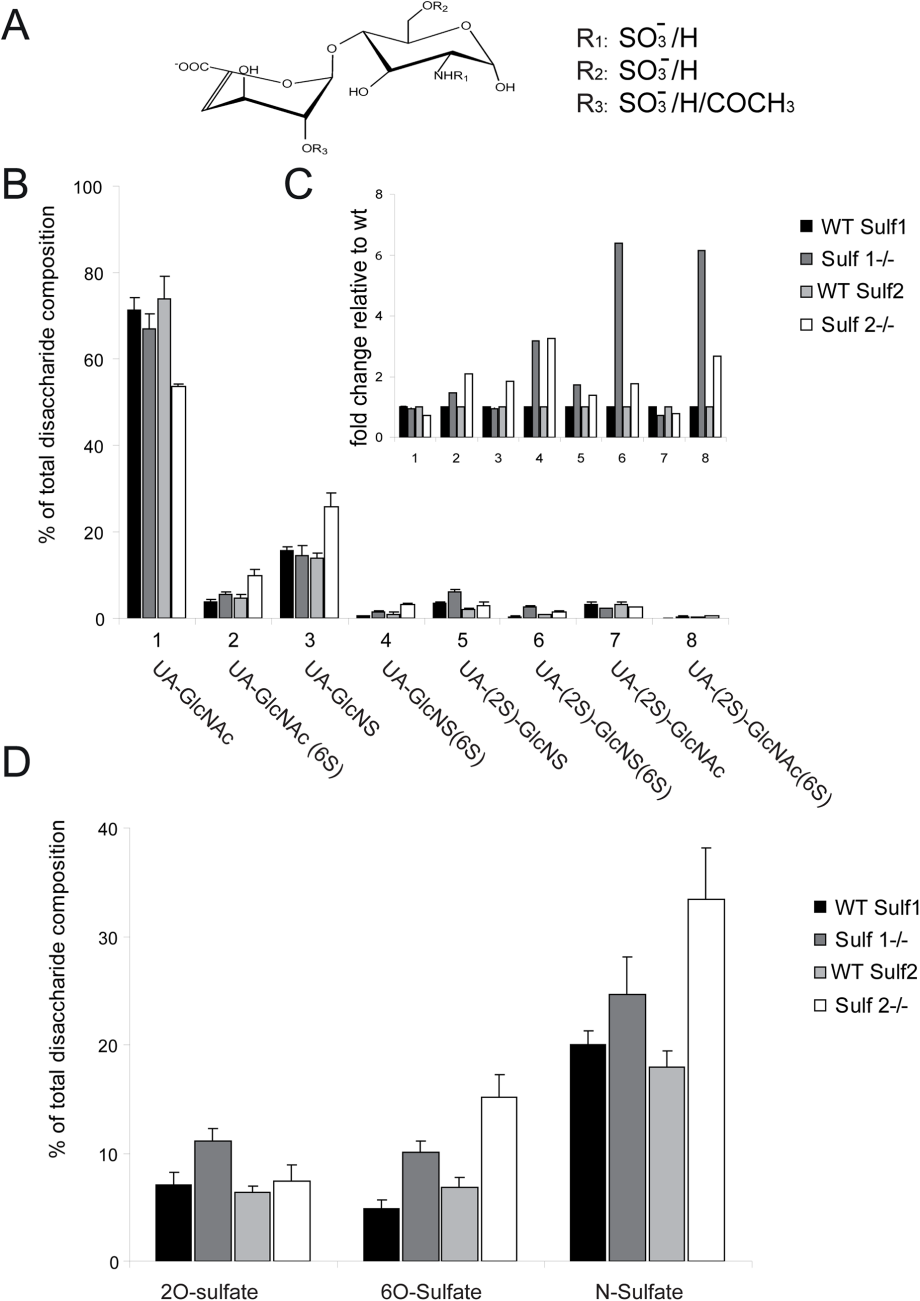
Disaccharide compositional analysis of HS from cerebella of postnatal day 6 Sulf deficient mice and wildtype littermates. HS from Sulf1 matched wildtype (black) and null (dark grey) as well as Sulf2 matched wildtype (light grey) and null (white) mice was purified using TRIzol, digested to disaccharides and labelled with BODIPY-FL hydrazide. Resulting disaccharides (with variant sulfation patterns depicted in panel A) were separated using strong anion exchange HPLC as described in Materials and Methods. Data are expressed in % of total disaccharide composition (**B**) and as fold change relative to wildtype (**C**) (mean +/- SD; n = 3, from individual cerebella). (**D**) Total NS, 2S and 6S containing disaccharides in postnatal cerebellum HS of Sulf deficient mice. Data are expressed in % of disaccharide composition. 1, ΔUA-GlcNAc; 2, ΔUA-GlcNAc(6S); 3, ΔUA-GlcNS; 4, ΔUA-GlcNS(6S); 5, ΔUA(2S)-GlcNS; 6, ΔUA(2S)- GlcNS(6S); 7, ΔUA-2S-GlcNAc; and 8, ΔUA-2S-GlcNAc(6S).

As described above, FGF2, GDNF and NGF signal transduction pathways are differentially affected in Sulf1 or Sulf2 deficient primary cerebellar granule cells. To link these effects with molecular changes in the sulfation of HSPGs (Fig 3A), which are the substrates of the Sulfs, HS was extracted from postnatal cerebella of Sulf1, Sulf2 and their age-matched wildtype littermates. Subsequently, the disaccharide compositional profile was analyzed by SAX-HPLC with high sensitivity fluorescence detection [35,36,37]. The compositional profiles of Sulf deficient cerebella revealed significant increases in all 6-O-sulfate containing disaccharides compared to control tissue (Fig 3B). Notably, there were clear Sulf-specific differences. In Sulf1 knockout mice cerebella, the HS displayed large increases in the disaccharides **4** [UA-GlcNS(6S)], **8** [UA(2S)-GlcNAc(6S)] and **6** [UA(2S)-GlcNS(6S)] (~3-, ~6- and ~6-fold increases, respectively, versus matched wildtype; Fig 3C). In contrast, in Sulf2 deficient mice the main increases were noted in disaccharides **2** [UA-GlcNAc(6S)], **8** [UA(2S)-GlcNAc(6S)] and **4** [UA-GlcNS(6S)] (~2-, ~3 and ~3 fold increases, respectively, versus matched wildtype) with only a small increase in **6** [UA(2S)-GlcNS(6S)] (~2-fold) (Fig 3C). These data indicate that Sulf2, compared to Sulf1, acts much less on the trisulfated disaccharide substrate *in vivo*, whereas both Sulfs act equally well on the disulfated disaccharide UA-GlcNS(6S). These findings further indicate that the two Sulfs do not (at least fully) compensate each other in case of single Sulf deficiency. In addition, we noted for Sulf1 deficient cerebella a small but significant increase of the 2-O and N-sulfated disaccharide **5** [UA(2S)-GlcNS] (Fig 3C). The overall changes in 2-O-, 6-O- and N-sulfation are shown in Fig 3D, demonstrating that the knockout of Sulf1 significantly (~2-fold) increases the amount of 2-O-sulfate groups in the postnatal cerebellum, whereas loss of Sulf2 enzyme has no influence on 2-O-sulfation. In contrast, in Sulf2 deficient postnatal cerebella a pronounced (~2-fold) increase of N-sulfation was observed (Fig 3D). This notable increase of N-sulfation in the Sulf2 knockout is mainly due to the N-sulfated disaccharide **3** [UA-GlcNS], the increase of which amounted to approximately 12% of total disaccharide composition (Fig 3B, S1 Table). These differences in sulfation, apart from 6-O-sulfation, obviously result from altered biosynthesis, probably as a consequence of feedback mechanisms due to the loss of Sulf activity [14,22]. In summary, the overall increase of 6-O-sulfated disaccharides clearly confirms the activity of Sulf1 and Sulf2 against 6-O-sulfate groups of HS. Furthermore, selective loss of Sulf1 or Sulf2 results in sulfatase-specific differences in sulfation patterns of HSPGs as shown here for the first time *in vivo*.

### Sulf deficiency alters HS specific epitopes of HSPGs at the cell surface of cerebellar granule cells

**Fig 4:**
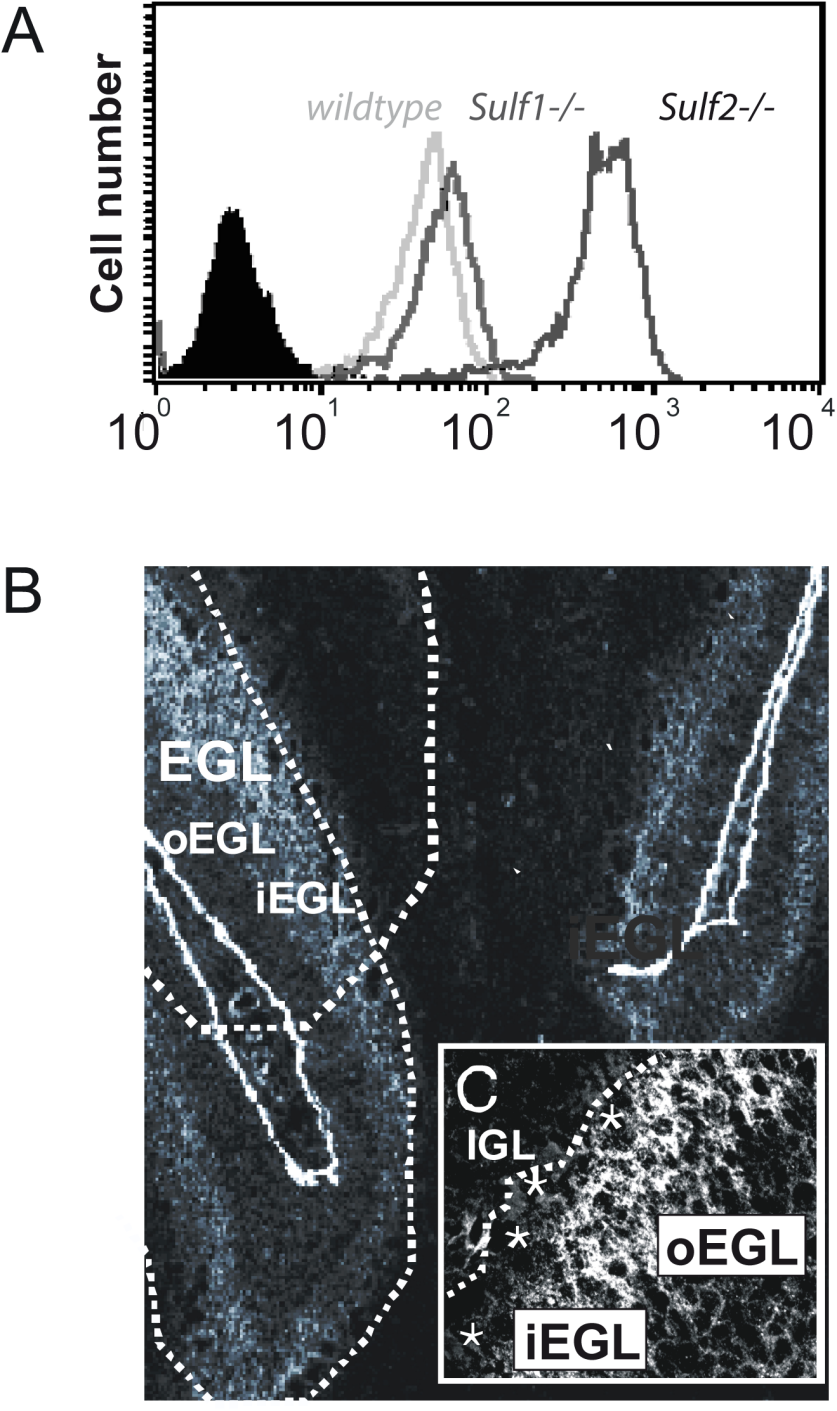
Increased binding of the EV3C3 ScFv antibody to HSPGs on the cell surface of Sulf2 deficient cerebellar neurons. (**A**) Cerebellar granule cells from wildtype (green), Sulf1 (red) and Sulf2 (blue) deficient mice were grown on culture dishes coated with a combination of PLL and laminin. Cells were harvested and incubated with the HS- specific phage display antibody EV3C3 and antibody binding was quantitated by FACS analysis. The binding profiles shown are representative of at least three different experiments. Control profiles (using the secondary antibodies alone) are shown as filled dark profile. (**B, C**) The HS specific antibody EV3C3 recognizes epitopes in the inner half of the EGL of the postnatal cerebellum. Cryosections from 6 day-old wildtype newborns were stained with the HS epitope specific antibody EV3C3 and fixed afterwards. The epitope recognized by the HS-specific ScFv antibody EV3C3 is present in a broad band of the inner EGL. (EGL: external granular layer, iEGL: inner EGL, oEGL: outer EGL, IGL: internal granular layer, asterisks: Purkinje cells, line: border between IGL and PCL) (B: 100 x, C: 630x).

We further analyzed the sulfate epitopes of HSPGs on the surface of primary cerebellar granule cells using the HS specific phage display ScFv antibodies HS4E4, RB4EA12 and EV3C3. FACS analysis using the antibody EV3C3 revealed a significantly higher binding to Sulf2 deficient compared to Sulf1 deficient and wildtype neurons (Fig 4A). EV3C3 recognizes an epitope defined by the presence of N-sulfation, epimerization and 2-O-sulfation. With respect to 6-O-sulfation, partial reduction of 6-O-sulfates increases reactivity of the antibody for heparin (T. H. van Kuppevelt, unpublished data), whereas complete 6-O-desulfation maintains strong reactivity [38]. This indicates a complex relationship between 6-O-sulfation and EV3C3 reactivity. Our data demonstrate a correlation between EV3C3 reactivity and elevated levels of the 6-O-sulfated Sulf2 substrate. In contrast, the RB4EA12 antibody, which binds to N-acetylated/N-sulfated domains that also contain 6-O-sulfates, as well as the HS4E4 antibody, preferring N-acetylated/N-sulfated oligosaccharides without 2-O and 6-O sulfates [39,40], showed no differences in binding to wildtype, Sulf1 and Sulf2 deficient neurons (data not shown).

HS can act as a guidance cue for outgrowing neurons and migrating cells. As the EV3C3 antibody allows detection of an alteration in the sulfation pattern of HSPGs on the cell surface of Sulf2 deficient neurons and, further, Sulf2-specific neurite outgrowth and cell survival deficits could be observed, we also analyzed the localization of the EV3C3-detectable HS epitope by immunofluorescence microscopy of cryosections of 6 day old cerebella, as shown in Fig 4B for the wildtype. The EV3C3 epitope is localized in a broad band tracing the inner EGL of the postnatal cerebellum, the region where precursor cells start to differentiate, in particular to migrate and build outgrowing neurites. No differences in the distribution of the epitope recognized by the EV3C3 antibody between cryosections from Sulf1 and Sulf2 deficient newborns and those of control mice were detectable (data not shown).

In summary, Sulf2 deficiency results in detectable differences in the sulfation patterns of HSPGs at the cell surface of cerebellar granule cells. Though detection of these sulfation pattern alterations by the EV3C3 antibody is only possible for isolated cells and not by immunohistochemistry on tissue sections, it is evident that the EV3C3 epitope histologically is localized in the developing cerebellum at postnatal day 6 in a broad band between the Purkinje cell layer and the outer EGL.

## Discussion

The cerebellum plays a central role in motor coordination, which is compromised in Sulf deficient mice [41]. Sulf2 mutants are characterized by impaired spatial learning, while Sulf1 deficient mice show reduced nocturnal perambulation [26]. Although the histoarchitecture of adult Sulf1 and Sulf2 deficient cerebella is indistinguishable from that of wildtype mice, Sulf1 and Sulf2 deficient cerebellar granule cells display a reduction in neurite length [26]. In a recent report it was shown that Sulf1 and Sulf2 are highly expressed in a number of adult neuronal tissues and experimental evidence was provided that Sulf1/2 are involved in proteoglycan dependent signalling leading to inhibition of neurite outgrowth from dorsal root ganglia [42]. Therefore, the hypothesis arose that the observed neuronal phenotypes in Sulf-deficient mice are caused by developmental deficits in formation of proper synaptic connections and guidance of axons to their final destination. To study the mechanisms behind the observed phenotypes we analyzed the importance of the 6-O-endosulfatases Sulf1 and Sulf2 for the postnatal development of the cerebellum.

### Sulf1 and Sulf2 act as neuroprotective and neurite outgrowth promoting enzymes during the postnatal development of the cerebellum

In addition to the previously described neurite outgrowth deficits [26], we show in this study that both Sulf1 and Sulf2 deficiency result in reduction of cerebellar cell survival *in vitro*. Thus, these enzymes have a cell protective function in the development for this part of the brain. The cell death of cerebellar granule cells in the EGL is a naturally observed phenomenon, which occurs in parallel to the massive proliferation of the EGL [43]. It was further demonstrated that postmitotic cerebellar granule cells undergo apoptosis, because they fail to establish proper synaptic contacts with other cerebellar precursors [44]. Whether the impaired neurite outgrowth contributes to the enhanced cell death in Sulf deficient cerebellar granule cells, or whether other mechanisms are responsible for this impairment remains to be clarified. Interestingly, the insensitivity of Sulf1 as well as Sulf2 deficient cerebellar cells to staurosporine-induced cell death is similar to what has been described for hepatocellular cancer as well as head and neck squamous cell carcinoma cell lines [29,30]. The apparent contradiction for Sulf deficient cerebellar granule cells, characterized by an enhanced apoptosis on the one hand and the insensitivity to induced cell death on the other hand, may point to Sulf function as a primary regulator of cellular signaling, deficiency of which is dominant over downstream staurosporine effects. Furthermore, based on the unaffected migration capacity of Sulf2 deficient neurons and the reduced number of migrated Sulf1 deficient cerebellar granule cells only on the non-stimulating-substrate PLL (mimicking an artificial control situation never present in the extracellular matrix *in vivo*) (Fig 1B), we conclude that the Sulf enzymes are less important for the migration of cerebellar granule cells. Since this process determines the correct lamination of the adult cerebellum, these results are in line with the observed unaltered histoarchitecture of Sulf1 and Sulf2 deficient postnatal cerebella as compared to wildtype controls (data not shown).

### Sulf deficiency results in sulfatase-specific alterations of HS structure *in vivo* and *in vitro*

The molecular origin leading to the observed Sulf specific phenotypes is to be found in differences of HSPG sulfation, which is modulated by the Sulf enzymes. Therefore, we analyzed the HS structures of cerebellar granule cells for Sulf-specific alterations using phage-display antibodies directed against specific HS epitopes. Interestingly, FACS analysis using the HS specific antibody EV3C3[45] indicated an increase of its cognate epitope on the cell surface of Sulf2 deficient cerebellar granule cells. Since HS from Sulf2 deficient cerebella has higher levels of 6-O-sulfate groups (Fig 3), this result suggests that the antibody might recognize the HS substrate structure of Sulf2, but not the reaction product. Therefore, antibody EV3C3 may be a useful tool to monitor Sulf2 activity. In addition, this observation may be due to the drastic increase of the N-sulfated disaccharide UA-GlcNS in Sulf2 deficient cerebella, although this needs further testing. The increase of this disaccharide (by approximately 12% of total disaccharide composition) was not seen in Sulf1 deficient mice (Fig 3 and S1 Table). These results lend further support to the already described hypothesis that the regulation of HS modifications is tissue- and developmental stage-specific [12,13,46,47], as it was formerly shown that the binding capacity of Sulf2 deficient embryonic fibroblasts towards the EV3C3 antibody was indistinguishable from that of Sulf1 deficient and control mouse embryonic fibroblasts [14]. Thus, Sulf deficiency influences the expression pattern of enzymes of the HS biosynthetic pathway, in particular the sulfotransferases, in a cell or tissue specific manner. Such a feedback mechanism affecting sulfotransferase expression was verified for Sulf deficient mouse embryonic fibroblasts [22], and can be inferred here from the *in vivo* HS analysis of Sulf1 and Sulf2 deficient cerebella (Fig 3). Certainly in FGF signaling 6-O-sulfation appears to play an exceptional role in mediating signaling response [48,49], consistent with our observations from embryonic fibroblasts of Sulf KO mice [22]. However, FGF2 receptor activation also depends on backbone N-sulfate and 2-O-sulfate moieties, so compensatory changes in N- and 2-O-sulfation, as we observed here particularly in the Sulf2 knock-outs, are highly likely to modulate growth factor responses. Profiling the expression of sulfotransferases along with HS structure-activity studies has provided evidence for complex regulation of HS biosynthesis *in vitro* [20,22,49]. Our data suggest that future studies *in vivo* should be very revealing in terms of the concerted regulation of multiple signaling pathways by HS, including potential feedback mechanisms controlling biosynthesis.

It should be pointed out that even though an extensive increase in sulfation of HS in particular on Sulf2 deficient cerebellar neurons was observed, the possibility that these molecules simply act as extracellular barriers because of their high negative charge, as shown for the polysialylated cell adhesion molecule NCAM [33], another polyanionic cell surface molecule, can be excluded; in control experiments heparin as well as HS, when added as competitors, reduced the axon outgrowth capacity of cerebellar granule explants independent of the genotype (and resulting HS structure) of the studied cells (S1 Fig).

In addition to the analysis of HS sulfation patterns *in vitro*, in this study we have demonstrated for the first time Sulf-specific differences in HS sulfation patterns *in vivo* by profiling the disaccharide composition of HS from the postnatal cerebellum. In previous studies Sulf-dependent differences have been observed in embryonic fibroblasts derived from Sulf-deficient mice [14,19,22]; however, it is noteworthy that these results from cells in culture differ in terms of the extent and nature of Sulf-dependent changes from those seen here *in vivo*. This is likely due to altered HS biosynthesis and Sulf activity under tissue culture conditions, which would not be unexpected due to profound differences in the cell environment *in vitro*; such differences have previously been observed in developing neuroepithelium [12,13,50]. This emphasizes the importance of *in vivo* studies in order to gain an accurate view of the role of HS structural variations in developmental regulation. The data presented here (Fig 3) further underline the important role of the Sulfs in post-biosynthetic remodelling of HS structure.

### A model for Sulf action in signaling regulation

**Fig 5:**
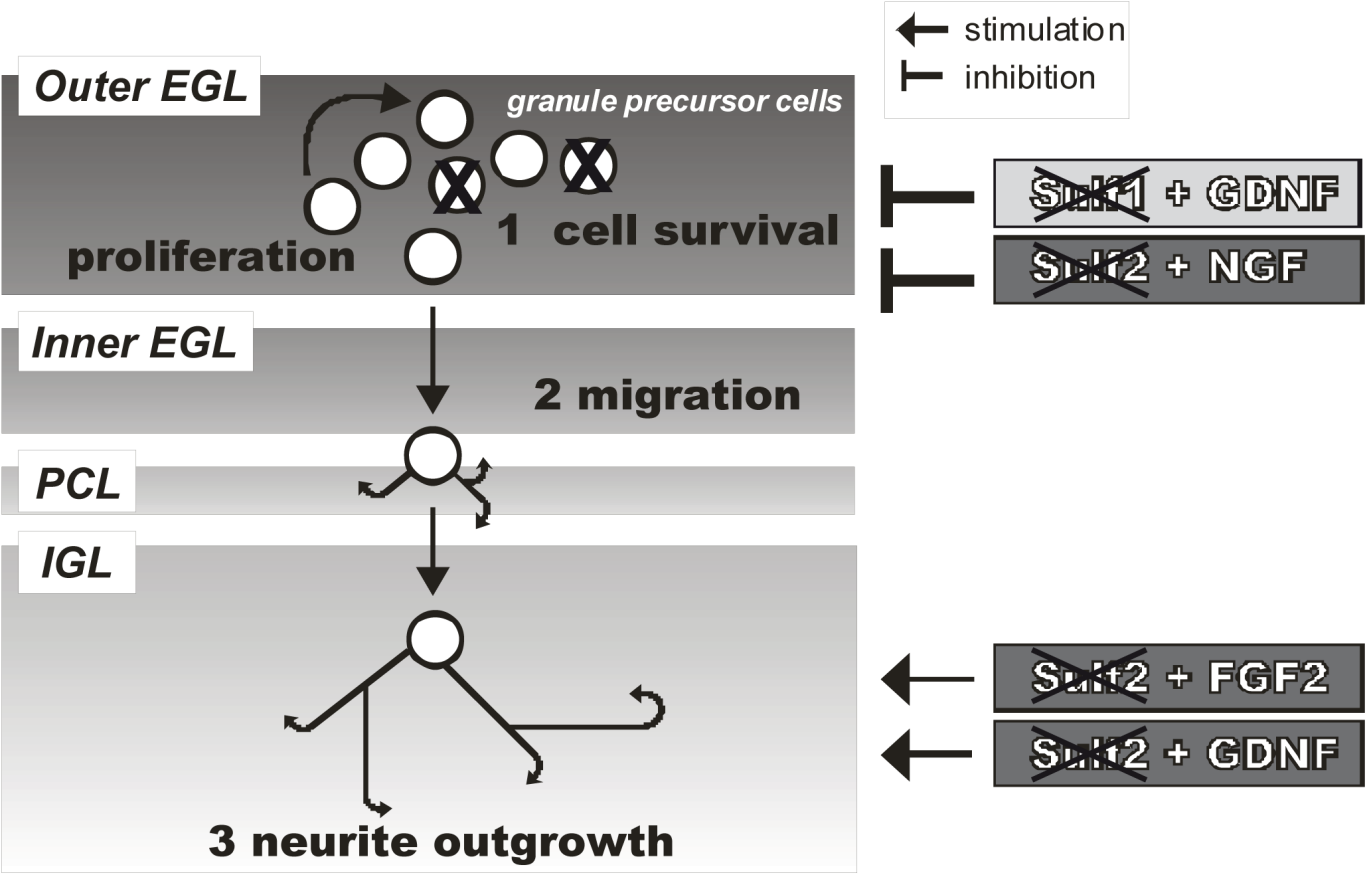
Sulf specific deficits during the postnatal development of the cerebellum are primarily mediated by altered signal transduction pathways. The outer half of the external granular layer (EGL) is the zone where precursors actively proliferate. Under the influence of GDNF, the cell survival of Sulf1 deficient precursor neurons is inhibited. A further proapoptotic effect was shown for NGF on Sulf2 deficient cells. Following the trail of a cerebellar precursor neuron, the cell enters the inner half of the EGL, stop dividing, and undergo neurite extension and tangential migration to reach their final destination in the internal granular layer (IGL) [1,2]. Neurite outgrowth deficits in Sulf2 knockout cells could be compensated by the addition of the growth factors FGF2 and GDNF, whereas no influence of growth factors such as FGF2, GDNF or NGF on neurite outgrowth was detectable for Sulf1 deficient neurons. The observed unaltered migration capacity (see Discussion) of Sulf deficient cerebellar granule cells is in line with inconspicuous cerebellar lamination in Sulf deficient mice.

In this study we show that Sulf deficiency results in altered responsiveness to the growth factors FGF2, GDNF and NGF, with specific effects observed for each sulfatase deficiency. According to these results, we propose a model explaining Sulf dependent deficits as a consequence of altered signal transduction pathways (Fig 5). In Sulf2 deficient neurons, FGF2 and GDNF promote neurite outgrowth, a step in postnatal development which occurs predominantly in the internal granular layer; by contrast Sulf1 deficient neurons fail to do so. Published observations made for Sulf1 and Sulf2 double deficient embryonic esophagus explants, which are characterized by a clear reduction in neurite length compensated by the addition of GDNF [19], are consistent with our results. However, although we observed a reduction in neurite length for both Sulf1 and Sulf2 deficient explants, only Sulf2 knockout neurons could compensate the deficit in the presence of GDNF (Fig 2A). Whether this observation is cell-type specific and/or developmental stage-dependent, or whether Sulf2 deficiency is “dominant” in the Suf1/2 double knockout, needs to be further clarified. On the other hand, GDNF protects cerebellar granule cells from cell death only in the presence of Sulf1 (Fig 2C, Fig 5). In addition, we observed a proapoptotic effect of NGF on Sulf2 deficient cerebellar granule cells (Fig 2B, Fig 5). This is surprising, since NGF has been postulated to act independent of HS [19,51]. Whether altered expression patterns of interacting receptors or downstream signaling factors of the NGF pathway contribute to the observed phenotypes is currently under investigation. Danesin and colleagues have shown that a reduction in Sulf1 expression inhibits Shh signaling [25]. It has also been shown that Sulf1 has a key role in regulating Shh-dependent neural cell diversity [23]. A role for Sulf1 in the ventral neural tube has also been described, whereby Sulf1 activity shapes the Shh morphogen gradient by promoting ventral accumulation of high levels of Shh protein [24]. Surprisingly, however, in our study signal transduction of Sulf deficient cerebellar granule cells in the presence of Shh, which is the “major coordinator” of growth and patterning of the cerebellum [5], is indistinguishable from that of wildtype neurons. This observation agrees with the unaltered histoarchitecture as observed in Sulf deficient cerebella and confirms the cell-type specific actions of the Sulfs.

## Conclusion

During the postnatal development of the cerebellum, the extracellular 6-O-endosulfatases Sulf1 and Sulf2 act as neuroprotective and neurite outgrowth promoting enzymes. The resulting HS code, with particular importance being attributed to 6-O-sulfate containing saccharide determinants, on the surface of neuronal precursors is required to orchestrate numerous underlying signal transduction processes. Sulf deficiency therefore could result in misguided neurons, deficits in proper synaptic connections and/or loss of neuron precursors, thus explaining the observed motor deficits in Sulf deficient mice [26]. How do the Sulfs, the only post-synthetic editors of the HS code, coordinate the integration of the various signals *in vivo*? Is there cellular crosstalk with other cells of the nervous system, like oligodendrocytes and astrocytes, which are also characterized by specific HS compositions [47]? The ‘quality control’ of the dynamic HS code in general and specific aspects of the role played by the Sulfs remain as open questions for further studies.

## Experimental Procedures

### Mouse husbandry and genotyping

Mouse lines were generated from knockout and wildtype littermates of Sulf1 and Sulf2 mutant mouse lines at postnatal day 6 with hybrid C57Bl/6 x 129 ola background, generated as described previously [14]. For determining the neurite length and cell survival of Sulf1 and Sulf2 deficient cerebellar neurons, however, due to tissue limitations for the statistical analysis, cultures were prepared using newborns from homozygous knockout breedings of Sulf1 or Sulf2 deficient mice. F1 hybrid newborns from C57Bl/6 and 129 ola intercrossings served as controls. In fact, preliminary experiments using either newborns of heterozygous intercrossings or homozygous breedings of the three described independent mouse lines led to the same results [26]. The mice were bred, housed and handled as approved by the local authorities (Gesundheits-, Veterinär- und Lebensmittelüberwachungsamt, City of Bielefeld, Az. 530.4 16 30 2). All animal care related to this study was carried out in full accordance with the German Protection of Animals Act.

### Microexplant *ex vivo* cultures

Microexplant cultures were prepared as previously described [26]. 16 h after plating, serum-free culture medium with or without the addition of heparin (Sigma-Aldrich, 10 μg/ml), HS (Sigma-Aldrich, 25 μg/ml), FGF2 (Sigma-Aldrich, 50 ng/ml), NGF (Sigma-Aldrich, 50 ng/ml) or GDNF (Sigma-Aldrich, 25 ng/ml) was added to the explants. After incubation for 24 h, the explants were fixed and stained. For neurite outgrowth analysis, the neurite length was quantitated by measuring the length of the ten longest neurites of fifteen aggregates in each experiment using the Leica IM1000 Image Manager (Leica, Wetzlar, Germany). For analysis of the migration of cerebellar granule cells, fixed explants were blocked for one hour with 0.1% BSA in PBS, stained with DAPI (Sigma-Aldrich), washed three times with PBS and mounted with Fluorescent mounting medium (DAKO Cytomation, Hamburg, Germany). Migration was quantitated by measuring the total amount of migrated cells of ten aggregates of three independent experiments using the AxioVision 4 Modul AutMess Plus software from Zeiss (Göttingen, Germany).

### Cerebellar granule cell culture

Cerebella from 6 day old wildtype, Sulf1 or Sulf2 deficient mice were dissected, washed with HBSS (PAA Laboratories, Cölbe, Germany) and digested with HBSS containing 1% Trypsin (Sigma-Aldrich), 0.1% DNase (Sigma-Aldrich) and 0.8 mM MgCl_2_ for 20 min. After digestion, cerebella were washed twice with HBSS, resuspended in HBSS containing 0.5% DNase and were triturated through a fire polished Pasteur pipette with reducing diameter. After centriguation for 10 min at 200 x *g* and 4°C, cells were resuspended in cell culture medium [MEM medium (PAA Laboratories) supplemented with 6 mM glucose, 200 μM L-glutamine (Invitrogen), 50 U/ml penicillin (Invitrogen), 50 μg/ml streptomycin (Invitrogen), 0.1% BSA, 10 μg/ml human transferrin (Sigma-Aldrich), 10 μg/ml insulin (Sigma-Aldrich), 4 nM L-thyroxine (Sigma-Aldrich), 0.027 TIU/ml aprotinin (Sigma-Aldrich) and 10 ng/ml sodium selenite (Sigma-Aldrich)].

### Analysis of cell survival

For the analysis of cell survival, 2.5 x 10^6^ cerebellar granule cells were plated into each well of a 48-well tissue culture plate, coated with poly-L-lysine (PLL) or a combination of poly-L-lysine and laminin, and cultured in cell culture medium. 16 h hours after seeding, cells were treated with growth factors GDNF (Invitrogen, 10 ng/ml), NGF (Invitrogen, 10 ng/ml) or FGF2 (Sigma-Aldrich, 10 ng/ml). After 24 h of culture, cell survival was determined. Control cells, i.e. without additives, were analyzed for each genotype; each control was set to 100%. For induction of cell death, staurosporine (500 nM; Sigma-Aldrich) was added to the medium 20 h after growth factor treatment. Viability of cells was assessed in all cases by counting the numbers of calcein- (Sigma-Aldrich) versus propidium iodide- (Sigma-Aldrich) positive cells. The cells from four randomly chosen areas of a microscopic field in each well were counted, and for each experimental value two wells were measured.

### Disaccharide composition analysis

HS was extracted and purified from individual cerebella in Trizol reagent (Invitrogen and [37]). The aqueous phase was applied to a DEAE column; HS was eluted with 2 M NaCl, and desalted on a PD10 column (GE Healthcare). Digestion reactions with recombinant heparinase enzymes (Ibex, Canada) from *Flavobacterium heparinum* were carried out at 37°C using initial digestion with heparinase I for 2 h, followed by heparinase III for 2 h and heparinase II overnight (16 hrs). Heparinase enzymes were used at 2.5mU in 10 μl heparinase buffer (100 mM sodium acetate, 0.1 mM calcium acetate, pH 7.0). HS disaccharides were labelled with Bodipy™ FL hydrazide (Invitrogen), separated by SAX-HPLC on a Propac PA-1 column (Dionex, UK), and detected in-line using a Shimadzu RF-551 fluorescence detector, as previously described [35,36,37]. Disaccharide peaks were identified by reference to the consistent elution positions of authentic disaccharide standards, and peak quantification performed using Shimadzu software with baseline subtraction, using previously calculated correction factors to account for differential labeling [52].

### FACS analysis

Cerebellar single cells cultured on laminin coated cell culture dishes were harvested using Versene (Invitrogen) and further analyzed by FACS analysis using the phage display antibodies EV3C3, HS4E4 and RB4EA12 as described previously [14].

### Immunofluorescence microscopy

Cryosections (5 μm) from 6 day old wildtype, Sulf1 or Sulf2 deficient newborns were hydrated with PBS for 10 min followed by a blocking step with 0.1% BSA in PBS for 20 min. Subsequently, the slices were incubated with phage-display antibodies (EV3C3, HS4E4 and RB4EA12, 1:1) for 1.5 h at room temperature. After three washing steps each for ten minutes with PBS, slices were incubated with the anti-mouse VSV secondary antibody (1:100; V-5507, Sigma-Aldrich, diluted in 0.1% BSA in PBS) for 1 h at room temperature. After three further washing steps with PBS and incubation with Alexa 488-coupled goat anti-mouse IgG (Invitrogen, 1:200, diluted in 0.1% BSA in PBS) for one hour at room temperature, slices were washed with PBS for three times, rinsed with methanol, dried and mounted with Fluorescent Mounting Medium (Dako Cytomation).

## Supporting Information

S1 Fig The inhibitory effect of heparin and HS on the neurite outgrowth of cerebellar explants is Sulf independent. Cerebellar microexplant cultures from wildtype (wt), Sulf1 (S1) and Sulf2 (S2) deficient mice were plated onto glass cover slips coated with PLL (P, filled bars) or a combination of PLL and laminin (L, hatched bars). Sixteen hours after plating, 10 μg/ml heparin (A) or 25 μg/ml heparan sulfate (B) were added to the medium. After incubation for further 24 h at 37°C, the explants were fixed and stained. Neurite outgrowth from the explants was quantitated by measuring the ten longest neurites of ten aggregates in three independent experiments. Neurite length of explants of each genotype cultured without any additives was set to 100%.
(TIF)

S1 Table Disaccharide composition of HS from cerebella of postnatal day 6 mice. Disaccharides were prepared, labeled with BODIPY-hydrazide and analyzed by HPLC with fluorescence detection as described in Materials and Methods. The proportions were corrected using the relative efficiency of BODIPY labeling for each of the disaccharide standards, as calculated previously [52]. Data are expressed in % of total disaccharide composition (mean +/- SD; n = 3, from individual cerebella).
(TIF)

## Abbreviations

DMEM: Dulbecco’s modified Eagle Medium
EGL: external granular layer;
FGF2: fibroblast growth facor 2
GlcA: glucuronic acid
Glc-NAc: N-acetylglucosamine
GlcNS: N-sulfoglucosamine
GDNF: glial derived neurotrophic factor
HBSS: Hank’s balanced salt solution
HS: heparan sulphate
HSPG: heparan sulfate proteolgycans, IdoA, iduronic acid
IGL: internal granular layer
iEGL: inner external granular layer
NGF: nerve growth factor
NS: sulfation
oEGL: outer external granular layer
PCL: Purkinje cell layer
PLL: poly-L-lysine
2S: 2O-sulfation
6S: 6O-sulfation
Shh: Sonic hedgehog
UA: hexuronic acid
wt: wildtype
