# Supplementary figures and images for: Sulf1 and Sulf2 Differentially Modulate Heparan Sulfate Proteoglycan Sulfation during Postnatal Cerebellum Development: Evidence for Neuroprotective and Neurite Outgrowth Promoting Functions
Source: PLoS One. 2015 Oct 8;10(10):e0139853. doi: 10.1371/journal.pone.0139853 (PMC4598108; doi:10.1371/journal.pone.0139853)

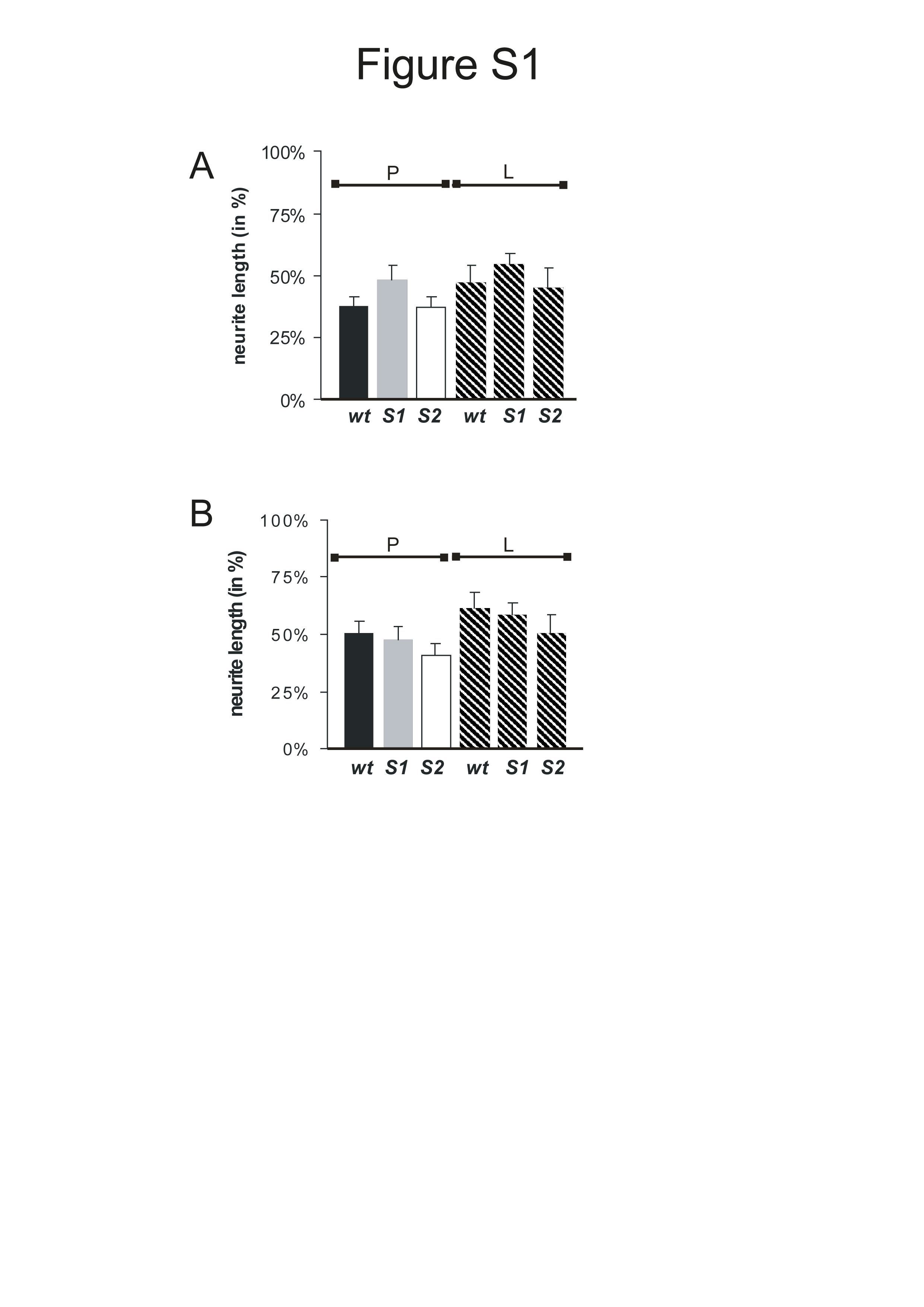

Supplement: S1 Fig — Cerebellar microexplant cultures from wildtype (wt), Sulf1 (S1) and Sulf2 (S2) deficient mice were plated onto glass cover slips coated with PLL (P, filled bars) or a combination of PLL and laminin (L, hatched bars). Sixteen hours after plating, 10 μg/ml heparin (A) or 25 μg/ml heparan sulfate (B) were added to the medium. After incubation for further 24 h at 37°C, the explants were fixed and stained. Neurite outgrowth from the explants was quantitated by measuring the ten longest neurites of ten aggregates in three independent experiments. Neurite length of explants of each genotype cultured without any additives was set to 100%. (TIF) [file pone.0139853.s001.tif]

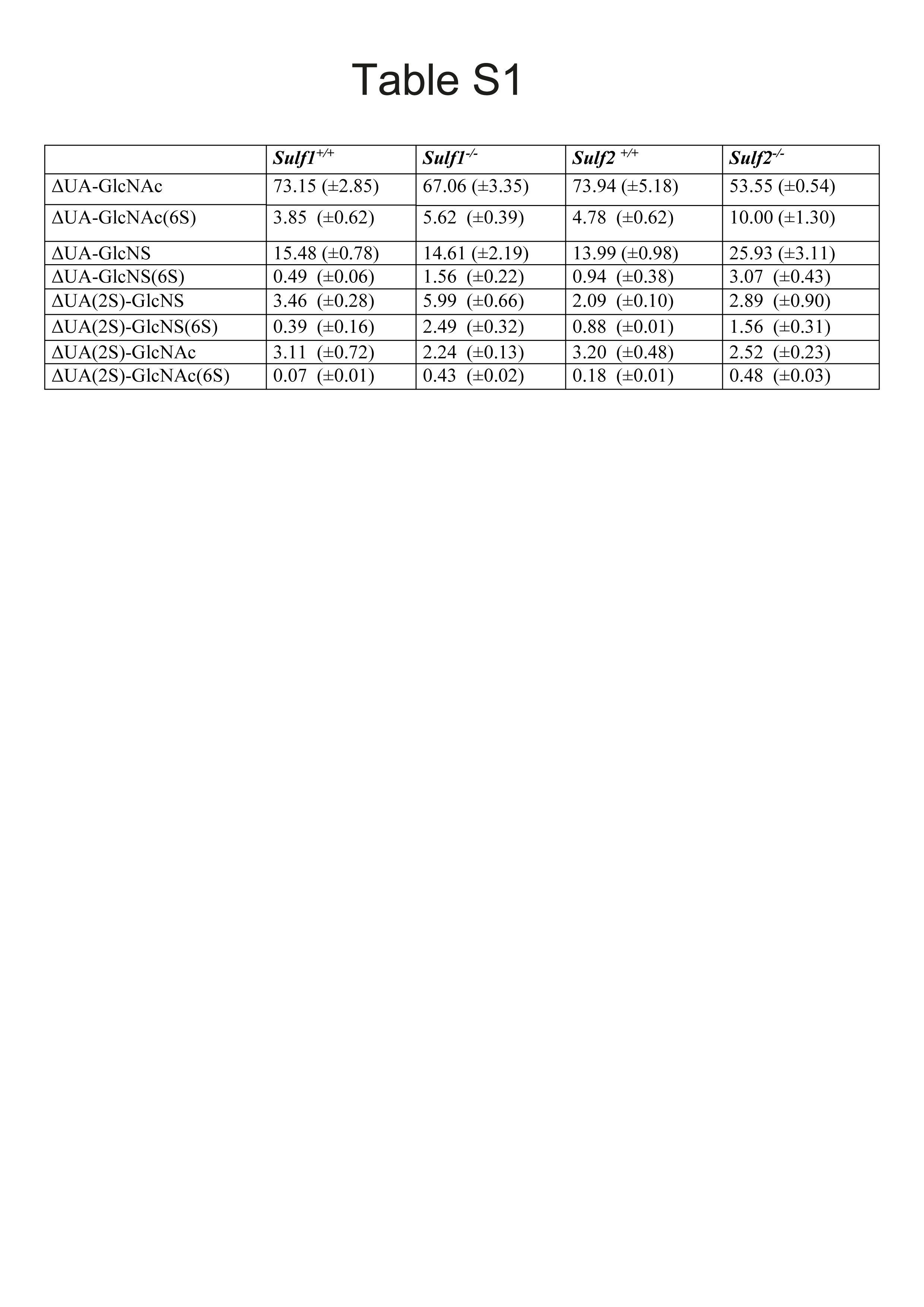

Supplement: S1 Table — Disaccharides were prepared, labeled with BODIPY-hydrazide and analyzed by HPLC with fluorescence detection as described in Materials and Methods. The proportions were corrected using the relative efficiency of BODIPY labeling for each of the disaccharide standards, as calculated previously [52]. Data are expressed in % of total disaccharide composition (mean +/- SD; n = 3, from individual cerebella). (TIF) [file pone.0139853.s002.tif]
